# Effects of human immunodeficiency virus and metabolic complications on myocardial nutrient metabolism, blood flow, and oxygen consumption: a cross-sectional analysis

**DOI:** 10.1186/1475-2840-10-111

**Published:** 2011-12-08

**Authors:** W Todd Cade, Dominic N Reeds, E Turner Overton, Pilar Herrero, Alan D Waggoner, Victor G Davila-Roman, Sherry Lassa-Claxton, Robert J Gropler, Pablo F Soto, Melissa J Krauss, Kevin E Yarasheski, Linda R Peterson

**Affiliations:** 1 Program in Physical Therapy, Washington University School of Medicine, 4444 Forest Park Boulevard, St. Louis, Missouri, 63108, USA; 2 Division of Geriatrics and Nutritional Science, Washington University School of Medicine, 660 Euclid Avenue, St. Louis, Missouri, 63110, USA; 3 Division of Infectious Disease, Washington University School of Medicine, 660 Euclid Avenue, St. Louis, Missouri, 63110, USA; 4 Cardiovascular Division, Washington University School of Medicine, 660 Euclid Avenue, St. Louis, Missouri, 63110, USA; 5 Division of Endocrinology, Metabolism, & Lipid Research, Washington University School of Medicine, 660 Euclid Avenue, St. Louis, Missouri, 63110, USA; 6 Division of Biostatistics, Washington University School of Medicine, 660 Euclid Avenue, St. Louis, Missouri, 63110, USA; 7 Department of Radiology, Washington University School of Medicine, 660 Euclid Avenue, St. Louis, Missouri, 63110, USA

**Keywords:** insulin resistance, cardiac metabolism and function, PET-imaging

## Abstract

**Background:**

In the general population, peripheral metabolic complications (MC) increase the risk for left ventricular dysfunction. Human immunodeficiency virus infection (HIV) and combination anti-retroviral therapy (cART) are associated with MC, left ventricular dysfunction, and a higher incidence of cardiovascular events than the general population. We examined whether myocardial nutrient metabolism and left ventricular dysfunction are related to one another and worse in HIV infected men treated with cART vs. HIV-negative men with or without MC.

**Methods:**

Prospective, cross-sectional study of myocardial glucose and fatty acid metabolism and left ventricular function in HIV+ and HIV-negative men with and without MC. Myocardial glucose utilization (GLUT), and fatty acid oxidation and utilization rates were quantified using ^11^C-glucose and ^11^C-palmitate and myocardial positron emission tomography (PET) imaging in four groups of men: 23 HIV+ men with MC+ (HIV+/MC+, 42 ± 6 yrs), 15 HIV+ men without MC (HIV+/MC-, 41 ± 6 yrs), 9 HIV-negative men with MC (HIV-/MC+, 33 ± 5 yrs), and 22 HIV-negative men without MC (HIV-/MC-, 25 ± 6 yrs). Left ventricular function parameters were quantified using echocardiography.

**Results:**

Myocardial glucose utilization was similar among groups, however when normalized to fasting plasma insulin concentration (GLUT/INS) was lower (p < 0.01) in men with metabolic complications (HIV+: 9.2 ± 6.2 vs. HIV-: 10.4 ± 8.1 nmol/g/min/μU/mL) than men without metabolic complications (HIV+: 45.0 ± 33.3 vs. HIV-: 60.3 ± 53.0 nmol/g/min/μU/mL). Lower GLUT/INS was associated with lower myocardial relaxation velocity during early diastole (r = 0.39, p < 0.001).

**Conclusion:**

Men with metabolic complications, irrespective of HIV infection, had lower basal myocardial glucose utilization rates per unit insulin that were related to left ventricular diastolic impairments, indicating that well-controlled HIV infection is not an independent risk factor for blunted myocardial glucose utilization per unit of insulin.

**Trial Registration:**

NIH Clinical Trials NCT00656851

## Background

Individuals infected with the human immunodeficiency virus (HIV) are at a greater risk for cardiovascular disease [1], myocardial infarction [2], and left ventricular dysfunction [3-5] than the general population. Although morbidity and mortality from HIV-related immune disorders have markedly declined [6], HIV-related cardiovascular disease has increased, signaling a new health crisis in the HIV-infected population.

HIV infection and combination anti-retroviral therapy (cART) directly impair peripheral nutrient metabolism [7,8] however their effects on myocardial metabolism and their relationship to left ventricular dysfunction are unknown. Approximately 50% of HIV-infected people treated with cART develop a cluster of peripheral metabolic complications (MC) [9] that include traditional cardiovascular disease risk factors such as dyslipidemia [10-12], peripheral insulin resistance [13-15], elevated blood pressure [16] and abdominal adiposity [17]: all components of "the metabolic syndrome" [18]. HIV infection/cART [19] and metabolic syndrome [20] also are associated with a pro-inflammatory state which further increases cardiovascular disease risk.

HIV-negative individuals with the metabolic syndrome develop left ventricular systolic and diastolic load independent abnormalities indicating that MC adversely affects left ventricular function [21]. In addition, HIV-negative individuals with MC have elevated myocardial fatty acid oxidation rates [22,23] and lower myocardial glucose utilization rates [22,24-27] and cardiac efficiency (i.e. ATP generation/oxygen consumed) [28]. cART components, especially nucleoside reverse transcriptase inhibitors, are associated with a myocardial mitochondrial toxicity [29] that may impair myocardial fatty acid metabolism in HIV+ patients. Also, HIV protease inhibitor-based cART has been associated with impaired peripheral fatty acid [8] and glucose (i.e. insulin resistance) metabolism [30,31]. The combination of impaired myocardial fatty acid metabolism (or decreased cardiac efficiency) and reduced myocardial glucose utilization, especially in the presence of metabolic inflexibility [32,33], may result in an impaired ability to generate ATP for contraction [34]. Although impairments in myocardial glucose metabolism may result in both systolic and diastolic contractile abnormalities [35,36], disruptions in myocardial glucose metabolism may manifest more through diastolic function due to the importance of ATP in cross bridge cycling, specifically during relaxation [37]. This is important as mild abnormalities in diastolic function frequently lead to overt heart failure later in life [38]. In addition, impaired myocardial glucose utilization may limit the heart's tolerance to ischemia, impair cardiac energetics and function [39,40] and predict worse outcomes following myocardial infarction [41].

Whether or not HIV infection, cART, and peripheral MC alter myocardial metabolism and its relationship to left ventricular function is unknown. The purpose of this study was to examine myocardial glucose and fatty acid metabolism and their relationship to left ventricular function in HIV-infected and HIV-negative men with and without MC. Due to their potential adverse effects on mitochondrial function, peripheral insulin sensitivity, and body composition, we hypothesized that well-controlled HIV infection (and cART) would be associated with lower myocardial glucose utilization and worse left ventricular function. Also, we hypothesized that peripheral MC, regardless of HIV status, would be associated with lower myocardial glucose utilization and worse left ventricular function. Specifically, we hypothesized that HIV+ men (taking cART) with MC would have the lowest myocardial glucose utilization and LV function compared to HIV+ men without MC, and HIV-negative men with or without MC. We also hypothesized that regardless of HIV status; men with metabolic complications would have lower myocardial glucose utilization rates and worse left ventricular function than men without metabolic complications. Lastly, we hypothesized that lower myocardial glucose utilization rate would be associated with lower diastolic function. We found that men with metabolic complications that include peripheral insulin resistance, with or without well-controlled HIV infection, have altered myocardial glucose utilization per unit insulin and left ventricular relaxation and that these alterations appear to be interrelated.

## Methods

### Participants

HIV-infected participants were recruited from the AIDS (Acquired Immune Deficiency Syndrome) Clinical Trials Unit and Infectious Diseases Clinics at Washington University School of Medicine in St. Louis, Missouri, USA. Due to the complexity and expense of these imaging studies, phenotypic data obtained from HIV+ men were compared to the same obtained from HIV-negative men with and without MC from prior [28] and ongoing studies conducted at our Medical School. All inclusion/exclusion criteria were similar and experimental procedures were performed identically in both HIV+ and HIV-negative (i.e. controls) groups.

Volunteers were excluded if they were diabetic, taking beta-blockers or taking medications that affect lipid or glucose metabolism. Two HIV+ men with MC were taking an ACE inhibitor, one HIV+ man without MC was taking an ACE inhibitor and a diuretic. No HIV-negative men were taking any anti-hypertensive medications at the time of study. All men consumed < 3 alcohol-containing beverages/week, reported no use of recreational drugs or tobacco for at least 6 months prior to enrollment and were weight stable. HIV+ men (23 with and 15 without metabolic complications) were taking cART (for at least the past 6 months) that included: nucleoside reverse transcriptase inhibitors (MC+: 87% vs. MC-: 100%), nucleotide reverse transcriptase inhibitors (MC+: 48% vs. MC-: 40%), non-nucleoside reverse transcriptase inhibitors (MC+: 17% vs. MC-: 27%), protease inhibitors: (MC+: 57% vs. MC-: 60%), boosted ritonavir: (MC+: 43% vs. MC-: 40%), and an integrase inhibitor: (MC+: 4% vs. MC-: 0%). Volunteers were excluded if their screening plasma viremia > 55, 000 copies/mL, or if they had an AIDS diagnosis (defined as either CD4+ T-cell count < 200 cells/μL or current/past opportunistic infection). The Human Research Protection Office at Washington University School of Medicine approved the study and all men provided informed consent prior to participation.

Eligible men were categorized into one of four groups based on their HIV status and the presence of MC: 1) HIV+ with MC (HIV+/MC+), 2) HIV+ without MC (HIV+/MC-), 3) HIV-negative with MC (HIV-/MC+) and 4) HIV-negative without MC (HIV-/MC-). Metabolic complications (MC) were defined as peripheral insulin resistance/glucose intolerance (fasting plasma glucose 100-126 mg/dL OR fasting plasma insulin ≥ 13 μU/mL), AND ≥ 2 of the following criteria: 1) abdominal obesity (either a waist circumference ≥ 102 cm or BMI ≥ 30 kg/m^2^), 2) hypertriglyceridemia (fasting plasma triglycerides ≥ 150 mg/dL), 3) low high density lipoprotein (HDL)-cholesterol (fasting plasma HDL ≤ 40 mg/dL), or 4) elevated blood pressure (SBP ≥ 130 or DBP ≥ 85 mmHg; Table 1). These criteria represent a modification of the ATP-III definition for the metabolic syndrome [18]. We broadened the insulin resistance/glucose intolerance criteria because HIV+ people frequently have normal fasting glucose, but elevated fasting insulin levels [13,42]. Men with normal glucose tolerance and 1 or none of the above criteria were enrolled into a non-metabolically complicated group (i.e. HIV+/MC- or HIV-/MC-).

**Table 1:** Demographic, Body Composition, and Serum Metabolic Variables

Variable	HIV-/MC- (n = 22)	HIV-/MC+ (n = 9)	HIV+/MC-(n = 15)	HIV+/MC+ (n = 23)
Age (yrs)	25 ± 6	33 ± 5^†^	41 ± 6*	42 ± 6*
Median viral load (copies/mL)	NA	NA	UD (0-0)	UD (0-34, 000)
CD4+ T-cells (cells/μL)	NA	NA	585 ± 227	471 ± 202
HIV duration (months)	NA	NA	127 ± 80	113 ± 88
cART duration (months)	NA	NA	66 ± 54	72 ± 72
BMI (kg/m^2^)	24 ± 2	37 ± 2^‡^	24 ± 4	31 ± 5^§^
Waist circumference (cm)	82 ± 5	122 ± 9^‡^	84 ± 6	106 ± 13^§^
FFM (kg)	64 ± 7	78 ± 8	60 ± 10	70 ± 16
Fat mass (kg)	13 ± 6	37 ± 6^‡^	11 ± 5	24 ± 10^§^
Fasting glucose (mg/dL)	90 ± 5	100 ± 8^§^	86 ± 6	94 ± 8^¶^
Fasting insulin (μU/mL)	4.7 ± 2.7	20.2 ± 14.0^§^	4.8 ± 2.2	14.8 ± 6.0^§^
Fasting HOMA	1.1 ± 0.7	5.1 ± 3.7^§^	1.1 ± 0.5	3.4 ± 1.4^§^
TG (mg/dL)	90 ± 24	271 ± 117^§^	126 ± 69^†^	186 ± 89^†^
HDL (mg/dL)	51 ± 9	37 ± 8^†^	48 ± 13	38 ± 10^†^
LDL (mg/dL)	91 ± 21.1	108 ± 33^§^	87 ± 23	108 ± 28^§^
Chol (mg/dL)	160 ± 26	194 ± 38	159 ± 28	181 ± 36
Lactate (μmol/L)	857 ± 377	887 ± 464	572 ± 147	808 ± 282
FFA (μmol/L)	548 ± 254	690 ± 176	663 ± 178	591 ± 174

Data expressed as mean ± SD. UD = undetectable, cART = combination anti-retroviral therapy, BMI = body mass index, FFM = fat free mass, HOMA = homeostasis model of assessment for insulin resistance, TG = triglyceride, HDL = high density lipoprotein, LDL = low density lipoprotein, Chol = total cholesterol, FFA = free fatty acid, *: p < 0.05 vs. HIV-/MC- and HIV-/MC+, ^†^: p < 0.05 vs. HIV-/MC-, ^‡^: p < 0.05 vs. remaining groups, ^§^: p < 0.05 vs. HIV-/MC- and HIV+/MC-, ¶: p < 0.05 vs. HIV+/MC-.

### Experimental Procedures

Fat and fat-free mass were quantified using a Hologic Discovery (version 12.4; Waltham, MA, USA) enhanced-array dual-energy X-ray absorptiometer (DXA). Participants were admitted to the Clinical Research Unit at 1800 h the night prior to the study and were provided a standardized meal containing 12 kcal/kg body weight and 55% carbohydrate, 30% fat and 15% protein. At 1900 h, they ingested a high carbohydrate beverage (80 gm carbohydrates, 12.2 gm fat, 17.6 gm protein, Ensure; Ross Laboratories, Columbus, OH, USA) to ensure adequate muscle and hepatic glycogen stores. They were fasted overnight (12 hours). In the morning, an 18-gauge catheter was inserted into an antecubital vein for radiopharmaceutical infusion, and a second catheter was inserted into a contralateral hand vein (heated to 55°C) for arterialized venous blood sampling. To standardize for potential circadian variations, positron emission tomography (PET) imaging started at 0800 h [43] using a commercially available tomograph (Siemens ECAT 962 HR+, Siemens Medical Systems, Iselin, NJ, USA). Blood pressure and heart rate were monitored throughout the imaging study. PET imaging quantified myocardial blood flow after ^15^O-water injection, myocardial oxygen consumption (MVO_2_) after 1-^11^C-acetate injection, myocardial glucose extraction fraction and utilization (GLUT) after 1-^11^C-glucose injection, and fatty acid extraction fraction, utilization, oxidation and esterification after 1-^11^C-palmitate injection. All PET procedures have been previously described and validated [44-46]. During the PET, arterialized venous blood samples were obtained at predetermined intervals for plasma substrate (glucose, fatty acids, and lactate), insulin, and radio-labeled metabolite concentrations. Validated compartmental models were used to calculate myocardial substrate kinetic rates [45-47].

### Echocardiography

Immediately after PET imaging, a complete 2-D, Doppler and tissue Doppler echocardiographic examination was conducted (Sequoia Cypress, Acuson-Siemens, Mountain View, CA, USA). Left ventricular (LV) end-diastolic and end-systolic volumes and LV mass were determined according to recommendations of the American Society of Echocardiography [48]. Pulsed-wave Doppler mitral inflow velocities of early left ventricular filling (E) and atrial filling (A) were obtained at the mitral leaflet tips in the apical 4-chamber view for calculating E/A ratio. Tissue Doppler imaging was performed in the apical 4-chamber view to determine the peak systolic shortening velocity (S_m_) and early diastolic myocardial relaxation velocity (E_m_) for regional assessment of systolic and diastolic function, respectively. E_m _and S_m _were calculated by averaging the velocities of the lateral and septal base.

### Plasma Analyses

Plasma glucose concentration was measured using an automated glucose analyzer (Yellow Spring Instruments, Yellow Springs, OH, USA). Plasma insulin levels were quantified using a chemiluminescent immunometric method (Immulite; Siemens, Los Angeles, CA, USA). Peripheral insulin resistance was evaluated using HOMA-IR [49]. Fasting plasma lipid/lipoproteins were quantified as previously described [50]. Lactate concentration was measured using a colorimetric assay kit (Sigma Chemicals, St. Louis, MO, USA).

### Statistical Analysis

Statistical comparisons were performed using SAS version 9.1 (SAS^®^, Cary, NC, USA). Between group differences in continuous variables (myocardial substrate kinetics, left ventricular function) were determined using two-way analysis of covariance (ANCOVA) and post-hoc analysis by Tukey HSD. The ANCOVA was adjusted for age, because HIV+ men were older than HIV-negative men. An independent t-test was used to compare dichotomous variables.

Pearson's correlation coefficient was used for univariate analysis of continuous variables; (e.g., left ventricular function during diastole and myocardial glucose utilization per unit plasma insulin (GLUT/INS)). Linear regression was used for multivariable analysis. Multivariable models were built in a manual forward stepwise fashion, considering variables with p < 0.10 in univariate analysis, to determine the most significant predictors of the specified dependent variables. Interactions of final predictors with HIV status were also examined. A p-value < 0.05 was considered statistically significant.

## Results

### Demographics

The HIV+/MC+ men were 35% African American, 6% Asian Indian and 59% Caucasian, HIV+/MC- were 40% African American, 7% Hispanic and 53% Caucasian, HIV-/MC+ were 100% Caucasian and HIV-/MC- were 10% African American and 90% Caucasian. HIV+ groups had a significantly greater percentage of African American men than HIV-negative groups (Table 1). As expected, total fat content measured by DXA was greater in the groups with MC compared to the groups without MC, and total fat content in HIV-/MC+ was greater than all other groups (Table 1). Based on the DXA regional fat measures, 32% of HIV+ men had "lipoatrophy" (< 5 kg limb fat), 50% had trunk adiposity (top 10^th ^percentile of all HIV+ men), 12% had a "mixed" phenotype (both lipoatrophy and trunk adiposity), and 6% had normal fat distribution. Other demographic variables are reported in Table 1.

### Myocardial Glucose and Fatty Acid Metabolism

**Figure 1:**
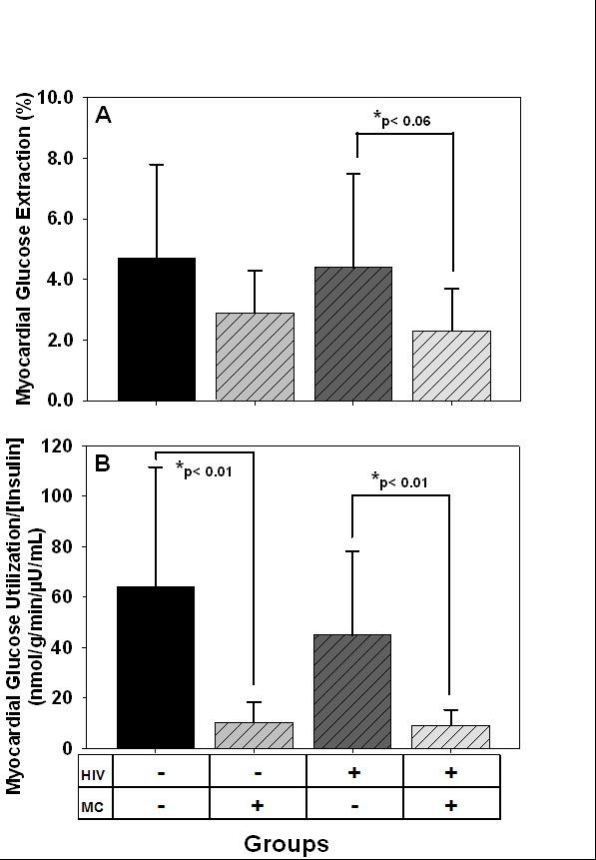
**(A) Myocardial glucose extraction fraction (%), and (B) Myocardial glucose utilization normalized to plasma insulin concentration (nmol/g/min/μU/mL) in HIV+ and HIV-negative men with and without metabolic complications (MC)**.

Myocardial blood flow and MVO_2 _were not different among groups (Table 2). There was a trend towards lower myocardial glucose extraction fraction (i.e. percentage of blood glucose extracted by the left ventricle of the heart) in HIV+/MC+ than HIV+/MC- (p = 0.06, Figure 1A). However, myocardial GLUT was not different among groups, and downstream myocellular metabolism of glucose: i.e. glycolysis, glycogen storage, oxidation, and lactate production was not different among groups (Table 2). After adjusting for the prevailing plasma insulin concentration, myocardial glucose utilization per unit insulin (GLUT/INS) was lower in metabolically complicated groups (i.e. HIV+/MC+ and HIV-/MC+) than in non complicated groups (i.e. HIV+/MC- and HIV-/MC-) irrespective of HIV status (Figure 1B). Myocardial GLUT/INS was not different between MC+ groups (HIV+ vs. HIV-negative), and not different between the MC- groups. Regardless of HIV, basal myocardial glucose utilization was blunted in the presence of elevated plasma insulin in men with metabolic complications that included peripheral insulin resistance (HOMA-IR).

**Table 2:** Myocardial Metabolic and Function Variables

Variable	HIV-/MC- (n = 22)	HIV-/MC+ (n = 9)	HIV+/MC- (n = 15)	HIV+/MC+ (n = 23)
MVO_2 _(μmol/g/min)	4.3 ± 1.0	4.5 ± 1.3	3.9 ± 1.1	4.3 ± 1.1
MBF (ml/g/min)	0.97 ± 0.19	1.06 ± 0.22	0.92 ± 0.26	0.97 ± 0.29
GLUT (nmol/g/min)	242 ± 141	139 ± 71	184 ± 135	113 ± 66
Glycolysis (nmol/g/min)	69 ± 54	40 ± 30	80 ± 89	81 ± 39
Glycogen formation (nmol/g/min)	147 ± 82	127 ± 57	118 ± 90	35 ± 34
Lactate production (nmol/g/min)	10 ± 11	5 ± 7	18 ± 35	6 ± 9
Glucose oxidation (nmol/g/min)	62 ± 43	34 ± 23	70 ± 67	33 ± 33
EF Total (%)	42 ± 8	38 ± 6*	49 ± 19	37 ± 8^¶^
FAOX (nmol/g/min)	133 ± 56	145 ± 44	115 ± 47	97 ± 38
FAOX/MVO_2_	30 ± 13	34 ± 11	32 ± 15	23 ± 9
FAEST (nmol/g/min)	19 ± 20	10 ± 11	35 ± 28	24 ± 23
FAUT (nmol/g/min)	110 ± 89	155 ± 43	149 ± 55	122 ± 40
HR (bpm)	57 ± 7	72 ± 10^†^	60 ± 14	62 ± 10
SBP (mm/Hg)	116 ± 12	134 ± 1^† ^	125 ± 20	127 ± 14
DBP (mm/Hg)	63 ± 10	73 ± 9	73 ± 11	74 ± 9
RPP (arbitrary units)	6269 ± 1737	9684 ± 1864^‡^	7488 ± 1775	7896 ± 1666^†^
LVM MM (g)	185 ± 29	230 ± 42^§^	185 ± 40	189 ± 41
EDV (mL)	125 ± 23	129 ± 18	101 ± 25	110 ± 24
ESV (mL)	52 ± 12	53 ± 13	39 ± 10	44 ± 13
EF (%)	58 ± 4	59 ± 6	61 ± 5	60 ± 8
LVET (ms)	319 ± 25	272 ± 17^‡^	313 ± 37	297 ± 29
S_m _(cm/s)	8 ± 1	8 ± 2	8 ± 1	8 ± 1
E wave (cm/s)	74 ± 15	59 ± 21	71 ± 14	66 ± 16
E/A ratio	2.1 ± 0.6	1.5 ± 0.5	1.7 ± 0.6	1.4 ± 0.5
E_m _(cm/s)	17.2 ± 1.9	12.1 ± 2.4^§^	13.9 ± 1.7	12.8 ± 2.0^†^
DT (ms)	178 ± 29	197 ± 21	198 ± 35	202 ± 48
IVRT (ms)	80 ± 13	74 ± 8	79 ± 8	80 ± 7

Data expressed as mean ± SD. MVO_2_: myocardial oxygen consumption, MBF: myocardial blood flow, GLUT: myocardial glucose utilization, EFTotal: myocardial fatty acid extraction fraction total, FAOX: fatty acid oxidation, FAEST: fatty acid esterification, FAUT: fatty acid utilization, LVM = left ventricular mass, LVMI: left ventricular mass index, EDV: end diastolic volume, ESV: end systolic volume, EF: ejection fraction, LVET: left ventricular ejection time, E_m_: average myocardial relaxation velocity during early diastole measured at the lateral wall and septal bases, DT: deceleration time, and IVRT: isovolumic contraction time. *: p < 0.05 vs. HIV-/MC- and HIV-/MC+, ^†^: p < 0.05 vs. HIV-/MC-, ^‡^: p < 0.05 vs. remaining groups, §: p < 0.05 vs. HIV-/MC- and HIV+/MC-, ¶: p < 0.05 vs. HIV+/MC-.

Myocardial fatty acid extraction fraction (i.e. percentage of fatty acids extracted from blood by left ventricle) was significantly lower in HIV+/MC+ and HIV-/MC+ than HIV+/MC- (Table 2) but was similar to HIV-/MC-. Myocardial fatty acid uptake, esterification, and utilization rates were similar among groups (Table 2).

### Left Ventricular Structure and Function

Rate pressure product (i.e. cardiac work) was significantly higher in MC+ groups than HIV-/MC-, and higher in HIV-/MC+ than HIV+/MC- (Table 2). However, cardiac efficiency (i.e. cardiac work/MVO_2_) was not different among groups. Myocardial relaxation velocity averaged at the lateral wall and septum during early diastole (E_m_) was lower in both MC+ groups than HIV-/MC-, and was lower in HIV-/MC+ than HIV+/MC- (Table 2).

### Analyses Using Traditional Metabolic Syndrome Criteria

The myocardial metabolism findings were similar regardless of whether we used the modified definition for MC or the traditional NCEP/ATP-III criteria for metabolic syndrome [18] (i.e. excluding fasting insulin and BMI criteria). Both HIV+ and HIV-negative men with ATP-III metabolic syndrome had significantly lower GLUT/INS than HIV-negative men without the metabolic syndrome, and both tended to be lower than HIV+ men without the metabolic syndrome (data not shown). In addition, myocardial relaxation velocity averaged at the lateral wall and septum during early diastole (E_m_) was significantly lower in both groups with ATP-III metabolic syndrome than HIV-negative men without the metabolic syndrome, and was significantly lower in HIV-negative men with ATP-III metabolic syndrome than HIV+ men without the metabolic syndrome (data not shown).

### Analyses Using Immunological Status

We [42] and others [51] have noted associations between CD4+ T-cell count and the presence of metabolic complications and cardiovascular events in HIV infected adults. So, we performed a sub-analysis (controlled for age) that examined myocardial nutrient metabolism and left ventricular function between HIV+ men with current CD4+ T-cell count ≥ 500 cells/μL and HIV+ men with CD4+ count ≤ 500 cells/μL, HIV+ men with CD4+ T-cell count ≤ 500 cells/μL had significantly lower (p < 0.05) GLUT/INS (16.4 ± 22.5) than HIV+ men ≥ 500 cells/μL (27.9 ± 29.9 nmol/g/min per μU/mL insulin). Myocardial fatty acid oxidation and utilization rates were similar between groups (data not shown). Myocardial relaxation velocity during diastole (diastolic function) averaged at the septum and lateral wall was not different between the HIV+ groups with low or high CD4+ T-cell counts.

### Correlations with Myocardial Glucose Metabolism Endpoints

To avoid multicollinearity among the many demographic variables, we chose BMI (over DXA- derived body fat parameters) and diastolic blood pressure (DBP; over systolic BP) for multivariable analyses used to predict GLUT/INS. Variables that remained in the multivariable model included BMI (β = -1.80, p = .025), DBP (β = -1.11, p = .011), and HDL-cholesterol (β = .097, p = .022) (model r^2 ^= 0.31). The dichotomous variable HIV (positive or negative) was not a significant predictor of myocardial GLUT/INS. Moreover, multivariable models that included HIV did not predict myocardial GLUT/INS better than models that did not include HIV. When two outlier values for myocardial glucose utilization (191.6 and 199.7 nmol/g/min) were excluded from model predictions, the same variables remained in the model, and parameter estimates were slightly smaller (BMI β = -1.59, p = .003; DBP β = -0.82, p = .005; HDL-cholesterol β = 0.87, p = .002; model r^2 ^= 0.43).

### Correlations with LV Structure and Function Endpoints

Like above, we used BMI and DBP in the multivariable model for myocardial relaxation velocity during early diastole (E_m_). Variables that remained in the model included BMI (β = -.003, p < .001), DBP (β = -.0007, p = .007), HIV (β = -.100, p < .001), and the interaction of HIV and BMI (β = .003, p < .001) (model r^2 ^= .57). Thus, when considering the interaction with HIV, only BMI was significantly associated with myocardial wall velocity during diastole (E_m_) in HIV-negative men (β = -.003, p < .001), but not in HIV+ men (β = -.0005, p = .35).

**Figure 2:**
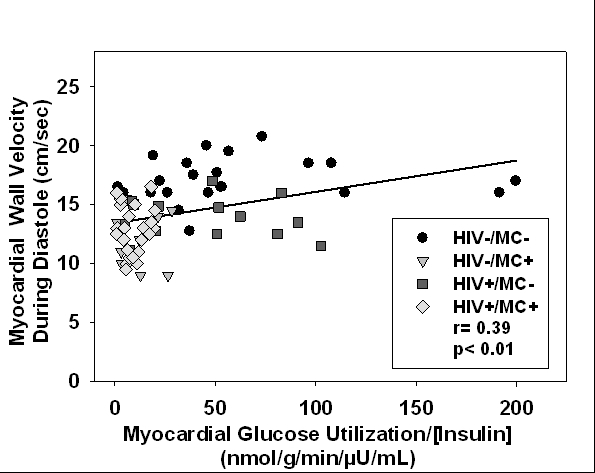
**Lower myocardial glucose utilization per unit plasma insulin (i.e. lower insulin sensitivity) predicted worse left ventricular diastolic function among all men**. MC = metabolic complications.

In univariate analyses, we examined the relationship between the two main dependent variables: GLUT/INS and myocardial relaxation velocity during early diastole. Myocardial GLUT/INS was significantly related to myocardial relaxation velocity during diastole (E_m_) (r = 0.39, p < .001, Figure 2), and related to filling velocity during early diastole (E/A) (r = 0.39, p = 0.001).

## Discussion

This is the first study to describe myocardial blood flow and nutrient metabolism in humans with HIV infection. The major findings are: 1) men with metabolic complications that included peripheral insulin resistance had lower basal myocardial glucose utilization rates per unit of fasting plasma insulin, irrespective of HIV status; 2) lower myocardial glucose utilization normalized to fasting plasma insulin was associated with worse left ventricular relaxation in both men with and without HIV; and 3) there were no differences in myocardial blood flow, MVO_2_, or myocardial fatty acid metabolism due to well-controlled HIV or metabolic complications. These findings imply that well-controlled HIV infection does not have an independent effect on myocardial metabolism in men. Regardless of HIV status, men with metabolic complications that included peripheral insulin resistance required a higher fasting plasma insulin concentration to achieve the same basal rate of myocardial glucose utilization as men without metabolic complications. This suggests that there is resistance to the ability of fasting insulin levels to mediate basal myocardial glucose utilization and this was associated with impaired left ventricular relaxation in men with metabolic complications.

We did not find that myocardial glucose metabolism and contractile function were worse in HIV+ men with metabolic complications than HIV-negative men with metabolic complications. The absence of the hypothesized effect may reflect the fact that these HIV+ men were virologically (low plasma viremia) and immunologically (CD4+ > 200 cells/μL) healthy. Perhaps myocardial metabolism/function is more adversely affected in men with more advanced HIV disease. In support of this notion, our sub-analysis indicated that HIV+ men with lower CD4+ T-cell counts had lower myocardial glucose utilization per unit insulin than HIV+ men with higher CD4+ T-cell counts. However, diastolic function (E_m_) was similar between these two HIV+ groups. These findings suggest important interactions between immune status and myocardial glucose metabolism (but not left ventricular diastolic function), that require further exploration.

Other potential explanations for the lack of a HIV effect on myocardial glucose metabolism and contractile function in men with metabolic complications include: the metabolic complications in the HIV+ men were less severe than those in the HIV-negative men. On average, HIV+ men had lower BMI, waist circumferences, and tended to have lower plasma insulin, glucose and triglyceride levels than HIV-negative men. If it were possible to perfectly match HIV-positive and -negative men on all traditional metabolic syndrome components, we may have found an additional adverse effect of HIV or cART on myocardial metabolism or function. This is practically very difficult, but could potentially be addressed in a larger study. Also, men in the current study were using contemporary cART regimens that may be associated with less metabolic complications than earlier cART regimens [52-54], and they may affect myocardial metabolism and function less. Also, the independent effects of cART on myocardial metabolism could not be sufficiently evaluated. HIV infected people naïve to cART are rare and may represent an inherently different group (e.g. long term non-progressors), and people who are initiating cART are difficult to capture and study due to their urgent treatment needs. In our study, the percentages of HIV+ men taking individual classes of cART were similar between those with and without metabolic complications. It is possible that specific cART drug classes affected myocardial metabolism differently, but this was not observed in the HIV+ men in our study. For example, HIV-protease inhibitors have been associated with cardiometabolic complications [11]. We compared myocardial metabolism and function between 23 men taking and 11 men not taking an HIV-protease inhibitor; both groups were taking similar nucleoside reverse transcriptase inhibitors. We found no differences or trends in myocardial metabolism or function between these two sub-groups (data not shown), suggesting that use of this cART class (protease inhibitors) does not account for the observed differences in myocardial metabolism and function. Importantly, this sub-analysis was underpowered, and we cannot confidently determine the independent effects of specific cART medications on myocardial metabolism/function.

**Figure 3:**
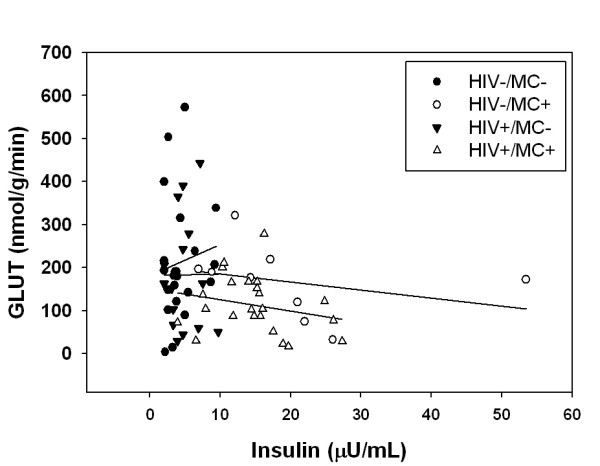
**Myocardial glucose utilization plotted against fasting plasma insulin concentration**. HIV-/MC- β = 7.36, HIV-/MC+ β = - 1.87, HIV+/MC- β = 0.37, HIV+/MC+ β = - 2.61.

We found that a higher fasting insulin concentration is required to achieve the same basal rate of myocardial glucose utilization in men with metabolic complications that includes peripheral insulin resistance. This agrees with studies conducted on HIV-negative people with type 1 and type 2 diabetes [23,25,26,55] who have reduced myocardial glucose utilization rates [25,26] and myocardial glucose utilization per unit insulin [23]. A plot of myocardial glucose metabolism versus fasting insulin concentration (Figure 3) generated lines with a negative slope for men with metabolic complications, while positive slopes were noted in men without metabolic complications. This implies that over the range of fasting insulin concentrations, men with metabolic complications did not increase myocardial glucose metabolism; this provides additional indirect evidence for basal myocardial insulin resistance. Although ideal, it is technically very challenging to conduct hyperinsulinemic-euglycemic clamps during myocardial PET studies, so a true dose-response curve for insulin was not obtained. Despite this limitation, we found that regardless of HIV status, the myocardium is capable of maintaining energy production from glucose utilization, but a higher fasting insulin concentration is required to achieve the same absolute myocardial glucose utilization rate. Impaired myocardial glucose utilization per unit insulin may adversely affect left ventricular function in men (with or without HIV) with metabolic complications. It may predispose men with metabolic complications to worse outcomes following a myocardial infarction or cardiovascular event since during ischemia, due to the decrease in oxygen availability, ß-oxidation of fatty acids decreases and subsequently the myocardium relies more on glucose to generate ATP [56,57]. Indeed, HIV+ men and women have a greater risk of mortality from myocardial infarction and chronic ischemic heart conditions than HIV-negative men and women [58]. However, without performing hyperinsulinemic clamp procedures in longitudinal outcome studies, the relationship between myocardial glucose metabolism and worse cardiac outcomes in HIV are only speculative. Also, decreased myocardial glucose utilization per unit insulin was not accompanied by a concurrent increase in myocardial fatty acid metabolism, suggesting that under some conditions (e.g. increased energy demand, ischemia) alternate fuel sources such as lactate, amino acids, or ketone bodies may be utilized to compensate for lower myocardial glucose utilization, and to achieve the myocardial ATP requirement. Myocardial lactate, amino acid or ketone utilization and their functional consequences need to be quantified in future studies.

We found that neither well-controlled HIV nor metabolic complications appeared to affect myocardial blood flow, MVO_2_, or myocardial fatty acid metabolism. This contrasts with findings from HIV-negative obese and non-obese women where these parameters increased with increasing BMI and/or peripheral insulin resistance [27]. The current findings are mostly consistent with those from HIV-negative men where there was no difference in myocardial blood flow and MVO_2 _between obese and non-obese, insulin resistant men [28]. In that study, myocardial fatty acid utilization was slightly higher in the obese men; this was primarily driven by higher serum free fatty acid levels in the obese, insulin resistant men [28]. We did not find significantly higher free fatty acid levels in men with metabolic complications, and consequently, their myocardial fatty acid metabolism was not different from men without metabolic complications. Interestingly, MVO_2 _among groups was similar even in the presence of increased cardiac work in the metabolically complicated groups suggesting that metabolically complicated groups appeared to be more efficient in generating energy per unit of MVO_2_; however, this was not a significant trend among groups (p = 0.10). Stress echocardiography/MVO_2 _could have also revealed differences in functional and metabolic reserve between groups. However, due to financial constraints, these parameters were not measured.

Among all the left ventricular function parameters, left ventricular relaxation was worse in the men with metabolic complications, regardless of HIV status. This left ventricular relaxation abnormality was not due to alterations in left ventricular macrostructure or systolic function. Our results appear to differ from those reported by Hsue et al. [5] who showed that HIV infection had an independent negative effect on diastolic function. However, there are substantial differences in participant characteristics between the two studies. Participants in Hsue et al. [5] were older (47 vs. 41 years), a greater proportion of participants were using illegal drugs (5% vs. 0%) or smoked tobacco (36% vs. 24%), were co-infected with hepatitis C (25% vs. 0%), and had prior coronary disease (5% vs. 0%). As a group, they had left ventricular hypertrophy, and a large proportion of participants had AIDS (median nadir CD4: 120) where no man in the current study had AIDS. The potential adverse effects of these worse co-morbid conditions on left ventricular function in the Hsue et al. study are difficult to ascertain because values for the components of the metabolic syndrome were not reported (only if participants were diagnosed as hypertensive, had hyperlipidemia, or had diabetes). It is possible that the presence of metabolic complications could obscure any potential detrimental effects of HIV infection on left ventricular function, but we hypothesized additive independent effects of HIV and metabolic complications on left ventricular function. This was not observed in the current study. Conversely, although diastolic function in men with peripheral metabolic complications was similar in the current study, multiple regression analysis revealed that HIV infection predicted lower diastolic function. This supports the findings from Hsue et al. [59] and others [5,60-62] in well-controlled or advanced HIV disease. But in these studies, the individual influence of metabolic complications was not considered. In the general population, metabolic complications adversely affect left ventricular systolic and diastolic function [21,63]. The current findings extend these and suggest that impaired diastolic function is associated with a reduced ability of fasting insulin to mediate myocardial glucose utilization in men with metabolic complications with or without well-controlled HIV infection.

Left ventricular diastolic function abnormalities are common, early findings in diabetic cardiomyopathy. The exact mechanism for peripheral and myocardial insulin resistance associated diastolic dysfunction is unknown, and it is unclear whether insulin resistance causes or is a marker of diastolic dysfunction. Previous animal research suggests that whole-body insulin resistance may cause: cardiac fibrosis [64], impaired myocardial calcium transport [65], advanced glycation end product-associated wall stiffness [66], and reduced fasting insulin-mediated myocardial glucose utilization [40]. The findings from our cross-sectional study can only suggest that dysregulated basal myocardial substrate utilization partially contributes to left ventricular diastolic dysfunction in HIV+ and HIV-negative men with metabolic complications. In multivariate analyses, BMI, DBP, and HDL-cholesterol (but not HIV status or HOMA-IR) were the strongest predictors for lower fasting insulin-mediated basal myocardial glucose utilization rate. The predictors were similar, even when HIV+ and HIV-negative groups were analyzed separately. Also, multivariate prediction models for diastolic function revealed that higher diastolic blood pressure was associated with worse diastolic function, and higher BMI was associated with worse diastolic function for HIV-negative men (but not HIV+ men). For men with low or normal BMI, HIV-negative men had better diastolic function than HIV+ men. This suggests that men with metabolic complications (regardless of HIV status) have a similar phenotype for myocardial glucose metabolism, but HIV infection per se may contribute (not additive) to left ventricular diastolic dysfunction. Our findings agree with those from HIV-negative people, where obesity and hypertension were associated with lower myocardial glucose utilization [28] and left ventricular diastolic dysfunction [67,68], and they support studies suggesting that HIV infection contributes to impaired diastolic function [59].

## Limitations

Due to the expense and the complexity of the PET studies, myocardial substrate kinetic rates obtained from HIV-negative men studied previously and in ongoing studies were used for comparison. Although the men in the study had similar metabolic profiles, hemodynamics, and left ventricular structure, on average, the HIV+ men were older than HIV-negative men. Thus, we adjusted for age in all analyses. Ideally, the race/ethnicity composition of the groups should have been equivalent, however, this has not been shown to affect myocardial metabolism [28]. Hyperinsulinemic-euglycemic clamps were not conducted during the myocardial PET studies, so the myocardium's responsiveness to increasing insulin levels was not determined. Basal measures of peripheral insulin resistance (HOMA-IR) in the absence of infused insulin correlate with peripheral glucose disposal rate measured during a glucose-insulin clamp (~r = 0.88) [69,70]. Our basal state findings may reflect or be intensified during a glucose-insulin clamp, but this requires further study. Our conclusions cannot be extended to other groups of people (women with HIV with/without metabolic complications or cART-naïve HIV+ people) [27], or those with measurable plasma viremia. We cannot rule out that HIV infection per se affects myocardial metabolism, as HIV infection itself affects peripheral metabolism [7]. Only 3 HIV+ men were taking anti-hypertensive medications, but the effects of ACE inhibitors and diuretics on heart metabolism is unknown and not likely to affect our conclusions. Also, large standard deviations in the myocardial metabolism data may have limited statistical power and precluded our ability to detect some differences in myocardial metabolism between groups. Biological variability in human myocardial substrate metabolism appears inherent as large variability in these measures has been previously reported previously [28,71,72]. Lastly, serum lactate levels appeared to be elevated in HIV-negative men without MC however we are unable to adequately explain this deviation.

## Conclusions

The findings suggest that men with metabolic complications that include peripheral insulin resistance, with or without well-controlled HIV infection, have altered myocardial glucose utilization per unit insulin and left ventricular relaxation. Moreover, these alterations appear to be interrelated. Impaired myocardial glucose utilization per unit insulin may detrimentally affect the heart's ability to adapt to conditions, such as ischemia, when the heart's reliance on glucose increases; this is speculative and requires further study. Neither HIV, nor metabolic complications, appears to affect myocardial blood flow, MVO_2_, or myocardial fatty acid metabolism. Further studies should determine if increasing myocardial glucose utilization per unit insulin improves left ventricular relaxation in people with metabolic complications.

## List of Abbreviations

A: late filling velocity during diastole measured by Doppler echocardiography; ACE: angiotensin converting enzyme; ANCOVA: analysis of covariance; ATP: Adult Treatment Panel; BMI: body mass index; cART: combination anti-retroviral therapy; DBP: diastolic blood pressure; DXA: dual energy x-ray absorbtiometry; E: early filling velocity during diastole measured by Doppler echocardiography; E_m_: average of lateral and septal wall velocity during early diastole measured by tissue Doppler echocardiography; GLUT: myocardial glucose utilization; GLUT/INS: myocardial glucose utilization per unit of plasma insulin; HDL: high density lipoprotein; HIV: human immunodeficiency virus; HOMA-IR: Homeostatic model of assessment- insulin resistance; MC-: metabolic complications negative; MC+:metabolic complications positive; MVO_2_: myocardial oxygen consumption; PET: positron emission tomography; SBP: systolic blood pressure; S_m_: average of lateral and septal wall velocity during systole measured by tissue Doppler echocardiography.

## Competing interests

The authors declare that they have no competing interests.

## Authors' contributions

WTC: study design; acquisition of data; analysis and interpretation of data; drafting and revision of manuscript, DNR: study design; analysis and interpretation of data; revision of manuscript, ETO: medical oversight; analysis and interpretation of data; revision of manuscript, PH: analysis and interpretation of data; revision of manuscript, ADW: acquisition of data; analysis and interpretation of data; revision of manuscript, VGD: analysis and interpretation of data; revision of manuscript, SLC: acquisition of data; revision of manuscript, RJG: study design; analysis and interpretation of data; revision of manuscript, PFS: acquisition of data; analysis and interpretation of data; revision of manuscript, MJK: analysis and interpretation of data; revision of manuscript, KEY: study design; analysis and interpretation of data; revision of manuscript, LRP: study design; analysis and interpretation of data; revision of manuscript. All authors approved the final version of manuscript
